# Targeted thermal stimulation and high-content phenotyping reveal that the *C*. *elegans* escape response integrates current behavioral state and past experience

**DOI:** 10.1371/journal.pone.0229399

**Published:** 2020-03-27

**Authors:** Jarlath Byrne Rodgers, William S. Ryu

**Affiliations:** 1 Department of Cell & Systems Biology, University of Toronto, Toronto, Ontario, Canada; 2 Donnelly Centre, University of Toronto, Toronto, Ontario, Canada; 3 Department of Physics, University of Toronto, Toronto, Ontario, Canada; McGill University, CANADA

## Abstract

The ability to avoid harmful or potentially harmful stimuli can help an organism escape predators and injury, and certain avoidance mechanisms are conserved across the animal kingdom. However, how the need to avoid an imminent threat is balanced with current behavior and modified by past experience is not well understood. In this work we focused on rapidly increasing temperature, a signal that triggers an escape response in a variety of animals, including the nematode *Caenorhabditis elegans*. We have developed a noxious thermal response assay using an infrared laser that can be automatically controlled and targeted in order to investigate how *C*. *elegans* responds to noxious heat over long timescales and to repeated stimuli in various behavioral and sensory contexts. High-content phenotyping of behavior in individual animals revealed that the *C*. *elegans* escape response is multidimensional, with some features that extend for several minutes, and can be modulated by (i) stimulus amplitude; (ii) other sensory inputs, such as food context; (iii) long and short-term thermal experience; and (iv) the animal’s current behavioral state.

## Introduction

As animals navigate their environment, they encounter various sensory information, which their nervous systems must process and integrate with their memory and internal physiological state to enact an appropriate response. This continuous coordinated response is what we consider behavior, and is the final output of an animal’s nervous system. Investigations of stereotyped escape behaviors or startle responses to various stimuli have provided extensive insight into the neuronal coordination of behavior in a number of model organisms, including flies, zebrafish, crayfish and molluscs [1–4]. However, how existing behavior affects the steps an animal takes to avoid incoming threatening stimuli, and how escape responses integrate past experience and information from other sensory systems has not been fully elucidated. The free-living nematode *Caenorhabditis elegans* is an attractive model organism for this work, because the full genome and connectome are known, and the full repertoire of body postures which comprise its behavior can be described with just four eigenworms [5–7].

Despite its relatively low-dimensional posture space, and apparently simple crawling behavior, new complexities in *C*. *elegans* locomotory behavior continue to be discovered. These discoveries were driven first by technological developments in the collection and processing of video behavioral data and, more recently, by progress in machine-vision based automatic behavioral quantification [8]. For example, since omega turns and reversals were first described as the primary *C*. *elegans* turning mechanisms [9], computer-aided analysis has made it possible to quantify at least two other reorientation behaviours: patterns of two or more turns in short succession, called pirouettes [10], and delta turns, large angle dorsal reorientations [11].

In addition to the description of new behaviors, higher throughput and more automatic analysis of behavior have made it possible to characterize locomotion phenotypes for hundreds of *C*. *elegans* mutant strains, in spontaneous and stimulus-evoked behavior paradigms [12,13]. Combining multiple behavioral metrics into behavioral barcodes or fingerprints has made it possible to identify previously undetectable phenotypes [14], and revealed that for thermal stimuli of different intensities, behavioral responses that appear qualitatively similar in fact rely on distinct genetic regulators [15].

When *C*. *elegans* is confronted with certain aversive stimuli, it executes a sequence of behaviors (collectively, an ‘escape response’) to reorient its locomotion away from the location of the stimulus. The classic escape response consists of a reversal, a deep turn to reorient direction, and resumed forward motion in the new direction, at an increased velocity [16,17]. *C*. *elegans* executes acute avoidance behaviors in response to a wide range of stimuli, including touch, changes in osmolarity, pheromones and noxious heat [16,18–21]. Although the responses to these myriad stimuli are collectively described as avoidance behaviors, and achieve the common goal of moving the worm away from the source of the noxious stimulus, it is important to note that a variety of behavioral strategies are employed. In this work, we used a focused infrared (IR) laser beam, steered by a pair of galvanometer-driven scanning mirrors, to target short (100 ms) pulses of heat at the worm’s head. Noxious heat avoidance behaviors can be triggered by crossing absolute temperature thresholds as well as by the rate of temperature change [22]. Our laser heating system induces *C*. *elegans* escape with relatively small (Δ*T* < 1.2 °C), but fast (*dT*/*dt* ≈ 1–5 °C/s) heating (S1B, S1C and S1D Fig).

Thermosensation in *C*. *elegans* is less understood than mechanosensation, but several neurons that respond to noxious heat have been identified, including AFD, AWC and FLP in the head, and PHC and PVD in the midbody and tail [23,24]. The AFD neurons are also required for sensing non-noxious temperature, and the related thermotactic behaviors essential to the survival of an ectotherm like *C*. *elegans* [25]. In addition to the sensory neurons involved in thermosensation, much of the neural circuitry involved in the escape response has been identified, at the cellular and molecular level [26,27]. However, although the neuronal connectivity is quite well characterized, an understanding of how sensory and motor signaling are coordinated to orchestrate complicated behaviors such as the escape response remains elusive [28].

In this work we aimed to expand our understanding of the experience- and behavioral state-dependence of the *C*. *elegans* response to noxious heat, by high-content quantitative phenotyping *C*. *elegans* escape behavior in a number of behavioral and sensory contexts. This approach required long-term tracking of individual animals at high resolution, and automatic targeting of a small infrared laser stimulus with variable amplitude. We fully quantified the resulting behavior by applying recent technological developments in posture decomposition and ethologically relevant behavioral metrics. Surprisingly, we found that the escape response is far more complicated than a simple stimulus-dependent reflex. Instead, *C*. *elegans* can modify its escape response depending on its long and short-term temperature experience, and its current behavioral state, demonstrating a high degree of plasticity in the response to similar stimuli despite a relatively simple neuronal circuit.

## Results

### System for long term tracking and targeted thermal stimulation of individual *C*. *elegans* combined with high-content behavioral phenotyping

**Fig 1:**
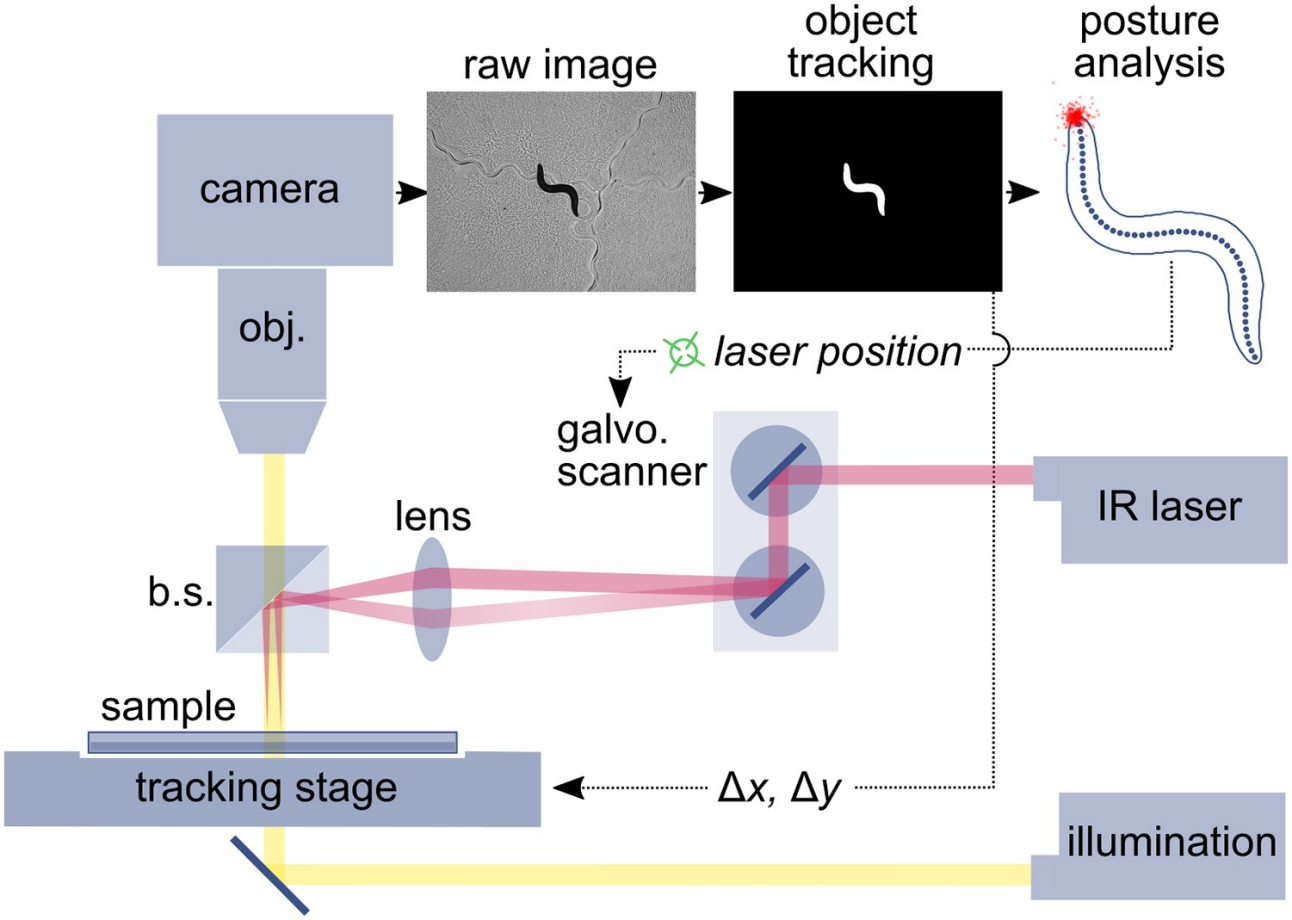
High spatiotemporal resolution thermal stimulation of freely behaving *C*. *elegans*. Worms crawl on an agar assay plate. The stage is manually moved by joystick to position a worm in the camera’s field of view, then the machine vision tracking system takes over automatic object tracking and laser targeting for the duration of the experiment. Dotted lines indicate flow of information in the system. The two red lines from ‘IR laser’ indicate two possible paths of the thermal stimulation beam, which is focused to a spot at the target position on the worm’s body. The red dots plotted around the head of the skeletonized worm indicate the laser locations for 392 stimuli. The worm’s body is approximately 1 mm long. See S1A Fig for a histogram of the head-stimulus distances. The yellow line from ‘illumination’ indicates the bright field illumination used to visualize the worm. Abbreviations: *obj*. = objective lens; *b*.*s*. = polarizing beam splitter; *lens* = 75 mm or 100 mm laser focusing lens; *galvo*. *scanner* = pair of galvanometer-driven scanning mirrors. See Methods for further details.

When a worm is confronted with noxious heat, or another aversive stimulus such as mechanical touch or vibration, it often responds with a sequence of behaviors that reorients its body and direction of movement away from the stimulus [16,29]. To investigate *C*. *elegans* escape behaviors in response to noxious heat, we developed a tracking microscope and infrared laser stimulation system, with precise and automated spatial control of an infrared laser stimulus that is small (full width at half maximum (FWHM) intensity = approximately 200 μm), targetable, and of scalable intensity and duration (Fig 1). Real-time machine vision software analyses the worm’s posture and location in each frame, allowing computer-controlled targeting of the stimulus. The software identifies the pixel location of the desired position along the worm’s body (in this work the worm’s head), and adjusts the scanning mirrors to appropriately steer the laser beam (Fig 1). The focused and steered IR stimulus is much smaller and more spatially and temporally precise than the stimuli used in previous studies of the *C*. *elegans* response to noxious heat, which include an electronically heated metal wire [19], heating or cooling a metal microscope stage [30], a thermal barrier many times larger than the worm [31], an area heated by an IR laser beam that the worm entered [19], and IR laser pulses with a beam diameter much larger than the worm’s body [15]. A system that is conceptually similar to ours has been used to activate temperature gated ion channels in the head and thorax regions of *Drosophila*, but we are able to achieve better spatial and temporal targeting [32].

From the high resolution video of the worm’s locomotion before and after the stimulus, we extracted its position and posture in each frame, and calculated a variety of metrics, including translational and phase velocity of the worm’s undulatory cycle [6], heading and position relative to the stimulus, and instantaneous behavioral state (forward, reverse, turn or pause). In their investigation of habituation of the escape response to photoactivation of the ASH neurons, Ardiel, E. et al. demonstrated the importance of considering not just the reversal response, but also changes in other components of ongoing behavior [33]. Similarly, we found it necessary to consider several behavioral metrics in order to describe differences in the escape response as it varied with the worm’s behavioral state, stimulus history and environment. In this work, three primary measurements are used to quantify the escape response: the fraction of the sample population occupying each behavioral state at each time point, phase velocity, and escape distance from the stimulus location. Combined, these measurements describe both the instantaneous behavior of the worm at each time point, and how that behavior results in changes in the worm’s position in the real world.

### Noxious thermal stimuli at the head elicit robust escape responses and induce a locomotory arousal state that lasts for several minutes

**Fig 2:**
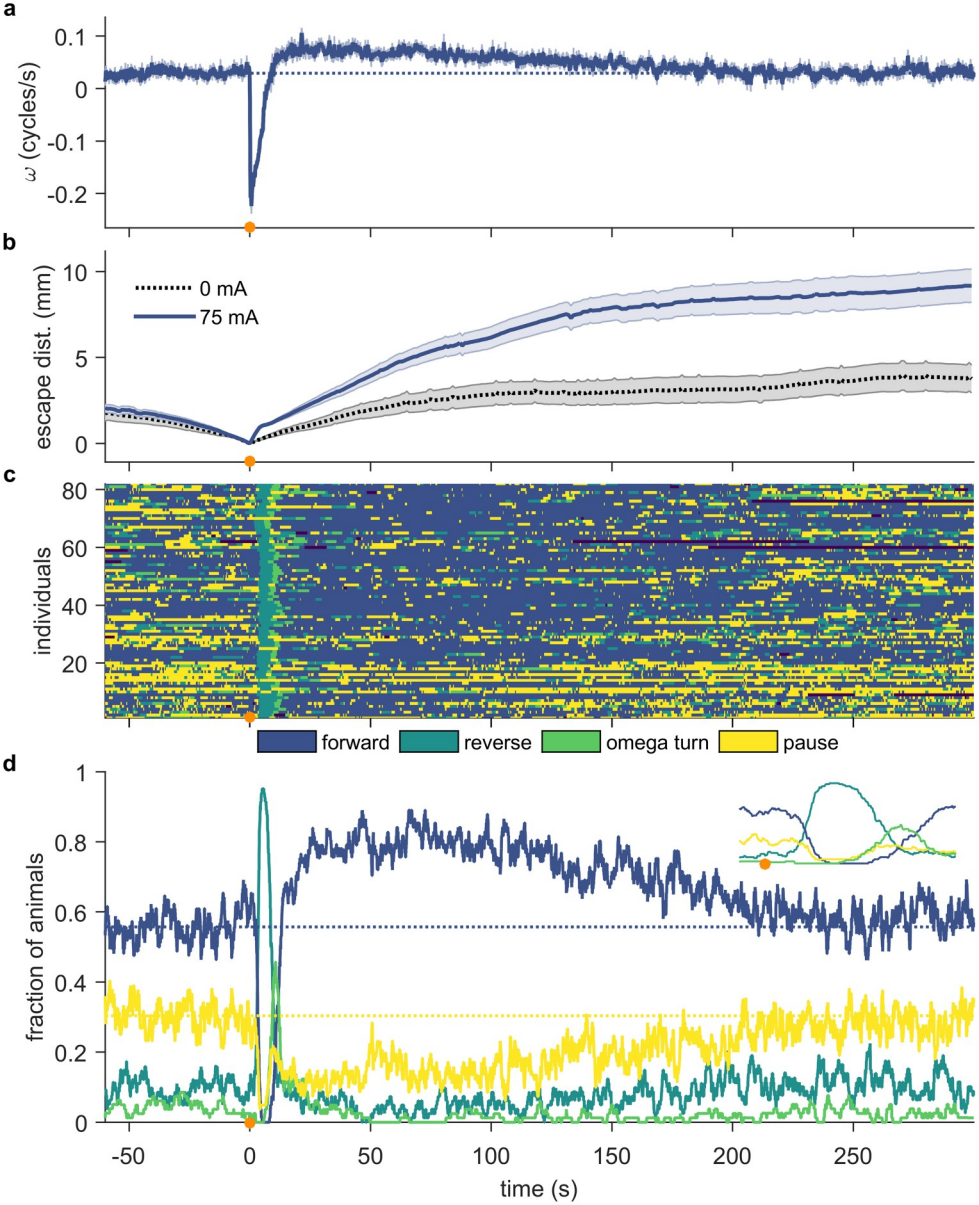
A single thermal stimulus elicits an immediate rapid escape response, and an extended period of locomotory arousal. The thermal stimulus is delivered by an IR laser (100 ms 75 mA pulse) targeted at the animal’s head (anterior 1/5th), and focused by a 75 mm lens. Plots are aligned so that the stimulus is at *t* = 0 s (indicated by an orange circle). (a) Mean phase velocity of the population of worms. Shaded region indicates the standard error of the mean. Note that the population’s average phase velocity takes greater than 180 s to return to pre-stimulus levels (the dotted dark blue line indicates the average pre-stimulus phase velocity). (b) Mean escape distance: the radial distance between the worm’s instantaneous centroid position and the centroid location at the time of the stimulus. The dotted black line shows data for unstimulated control animals. (c) Ethogram showing the instantaneous behavioral states of individual animals. One of four discrete states is assigned to each animal in each frame: forward, reverse, omega turn, or pause. (d) Fraction of animals in each behavioral state before and after the stimulus. The inset shows the period from 2 s before the stimulus until 15 s after the stimulus. Notice the well-described reverse-turn-forward sequence in the 15 s following the stimulus, indicated by the spike in reversal probability, followed by the spike in turn probability, and finally the elevated forward probability. The dotted dark blue and yellow lines indicate the average pre-stimulus fraction of the population occupying the forward and pause states, respectively. n ≥ 73 individuals.

This escape behavior has been considered highly stereotyped and short-lived, generally completed within 10 seconds [26]. However, certain stimuli cause far longer lasting changes in *C*. *elegans* behavior. In order to determine whether noxious heat can induce altered behavior over timescales of many minutes, we tracked single animals for five minutes after applying a focused IR laser pulse to the head. We confirmed that in response to noxious heat, similar to mechanical stimuli, the initial escape response prior to resuming forward motion is complete within approximately 10 s to 20 s. However, the population mean velocity remains elevated above the pre-stimulus level for approximately three minutes (Fig 2A), far longer than previously shown in response to noxious heat. We also considered how the distribution of behavioral states is affected by an encounter with a thermal stimulus. As expected, immediately after the stimulus, there is a significant increase in the fraction of animals in a reversal state (Fig 2C and 2D). As the escape response progresses, we observed the anticipated spike in the probability of a deep turn (*t* ≈ 10 s in Fig 2C and 2D). These increases correspond with decreases in the proportion of animals in the forward and pause state, which are near zero immediately after the stimulus. By 20 s post stimulus, most worms have resumed forward motion. However, the population’s distribution of behavioral states does not return to the pre-stimulus distribution until four minutes after the stimulus. Notably, forward probability remains elevated, while pause and turn probabilities and, to a lesser extent, reversal probability, are depressed. Combined with the initial escape trajectory, this shift in navigational strategy has the effect of biasing worms’ movement away from the location of the stimulus (Fig 2B).

### Stimulus strength affects both the probability and magnitude of the escape response

**Fig 3:**
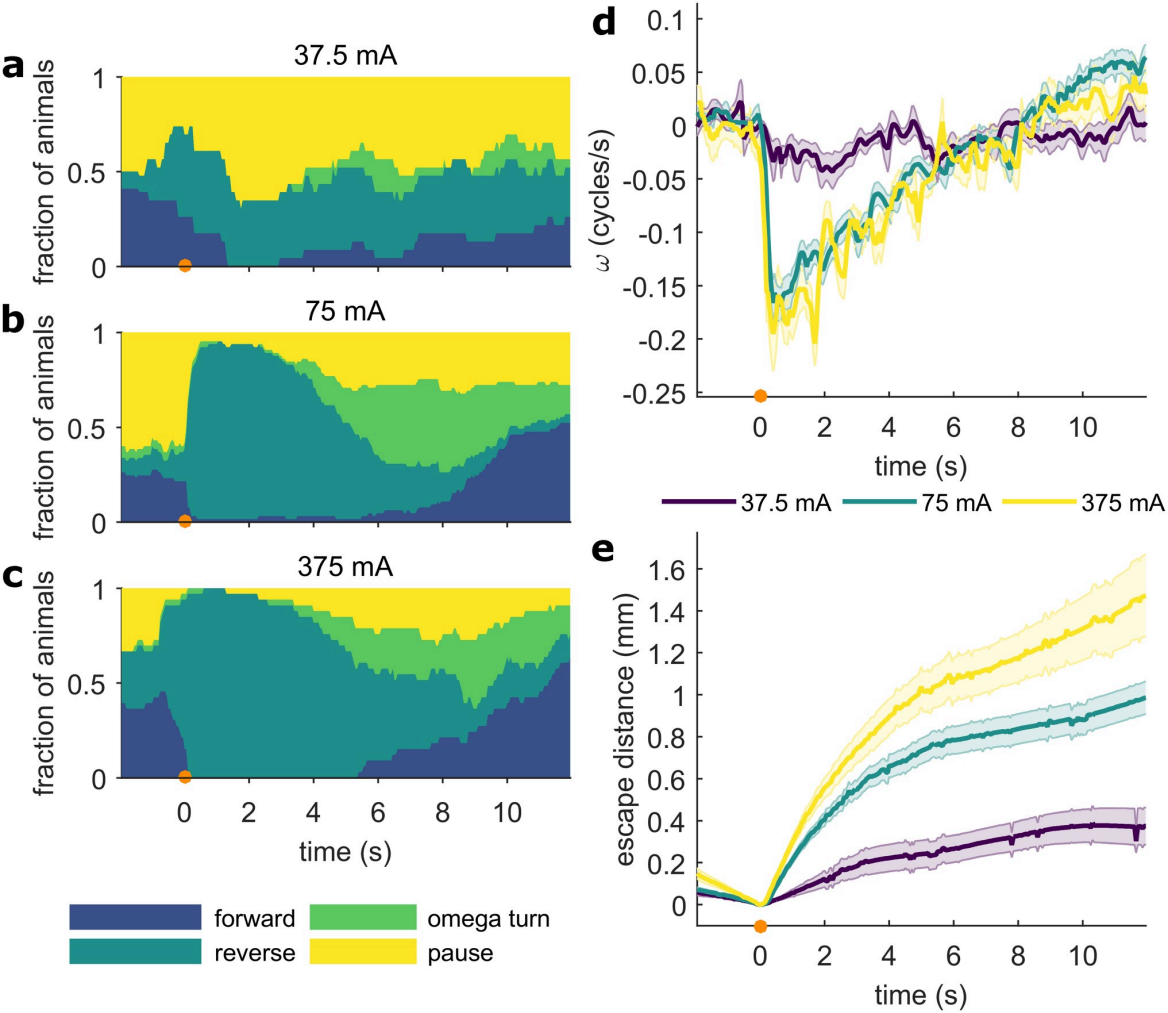
The probability and magnitude of the *C*. *elegans* escape response depends on the amplitude of the thermal stimulus. Responses to three levels of thermal stimuli are shown (all 100 ms, focused by a 75 mm lens). Plots are aligned so that the stimulus is at *t* = 0 s (indicated by an orange circle). (a-c) Fraction of animals in each of the four behavioral states, for three different laser power levels: (a) 37.5 mA (n ≥ 23), (b) 75 mA (n ≥ 65), and (c) 375 mA (n ≥ 33). 75 mA is the primary power level used in this work because it elicits robust responses from most animals, but does not fully saturate the response. (d) Population average phase velocity for each power level. Shaded regions indicate the standard error of the mean. (e) Mean escape distance. Shaded regions indicate the standard error of the mean. Full ethograms shown in S2 Fig.

We corroborated previous results indicating that at lower stimuli amplitudes, which correspond to smaller and slower increases in temperature (S1C and S1D Fig), the worm’s response becomes probabilistic [16]. For example, at 37.5 mA, some worms respond with a diminished escape reversal, others pause or slow down, and some appear to not respond at all (Fig 3A and S2A Fig). At 75 mA, the probability of an escape reversal increases dramatically, and most worms respond robustly with a strong reversal, often followed by an omega turn and accelerated forward motion (Fig 3B and S2B Fig). Compared to 37.5 mA, the reversal and omega turn probability is much higher in response to a 75 mA or greater stimulus (Fig 3A–3C). At 375 mA, the response probability saturates and 100% of worms execute a reversal after the stimulus (Fig 3C and S2C Fig). The *C*. *elegans* response to mechanical touch is also dose-dependent, although the response probability saturates at 80% [34,35]. In response to noxious heat, even when the response probability is saturated and 100% of worms reverse, other characteristics of the escape response, such as velocity (Fig 3D), reversal duration and probability of pausing, continue to depend on the amplitude of the stimulus (compare 75 mA and 375 mA in Fig 3B and 3C). Together, these results indicate that the worm is capable of a number of behaviors in response to different levels of noxious heat, and that both the probability and the magnitude of various response characteristics depend on the strength of the stimulus. Furthermore, modulating the magnitude of certain behavioral parameters such as velocity and reversal duration helps the worm escape more dangerous stimuli more efficiently (Fig 3E).

### Food context changes escape and baseline behaviors

**Fig 4:**
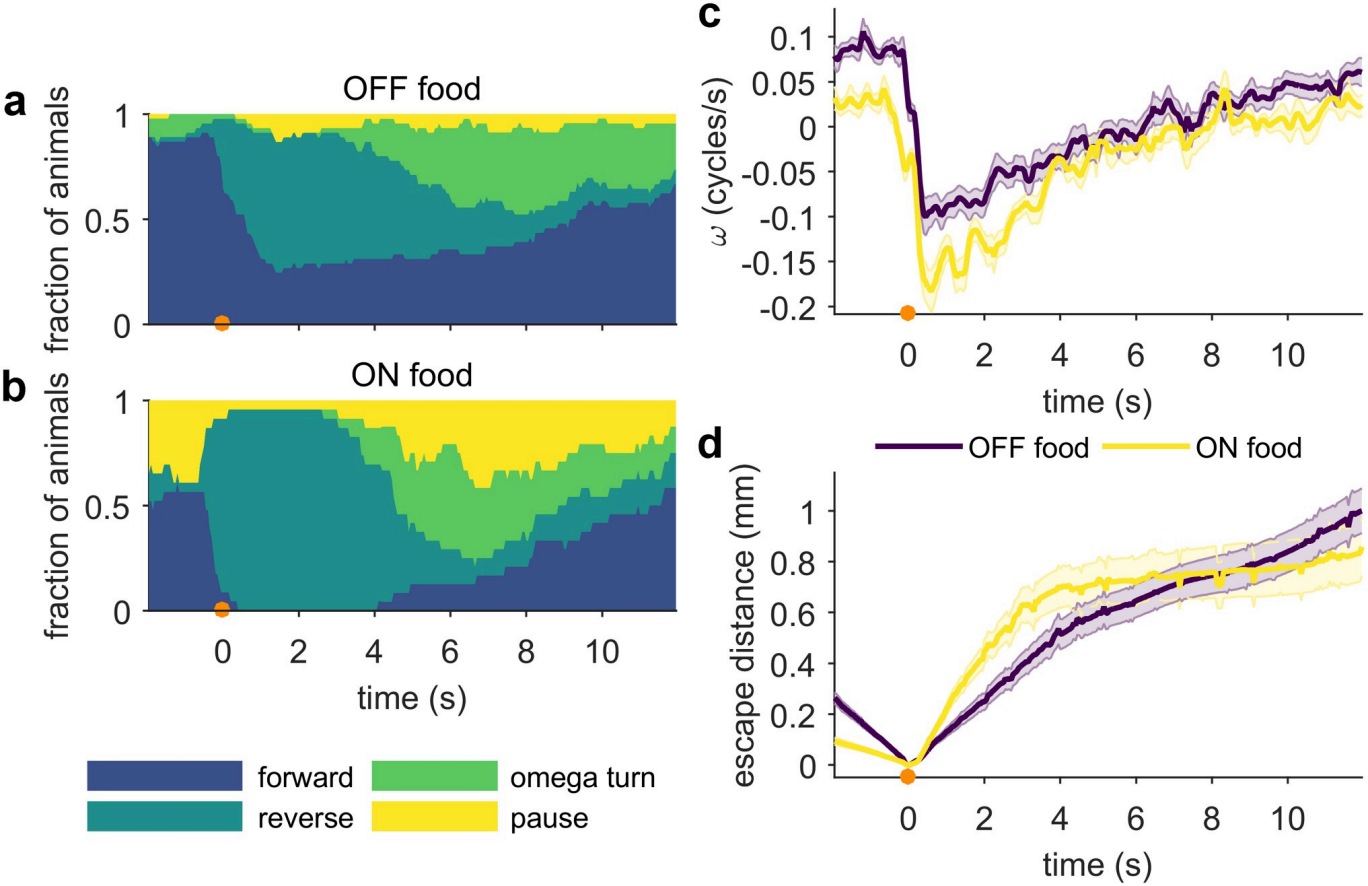
Escape reversals from noxious heat are less likely off food. Stimulus (100 ms 75 mA, focused by 75 mm lens) is at *t* = 0 s (indicated by an orange circle). (a-b) Fraction of animals in each behavioral state, OFF (n ≥ 46) and ON (n ≥ 24) food. Control ON food worms are washed with the same protocol as the OFF food animals. (c) Mean phase velocity. Shaded regions indicate the standard error of the mean. (d) Mean escape distance. Shaded regions indicate the standard error of the mean. Full ethograms shown in S3 Fig.

It is well documented that *C*. *elegans* basal locomotory behavior depends on its environment and sensory context, in particular food, one of the primary drivers of behavior [36,37]. However, how current food context affects noxious heat avoidance is unknown. To determine whether food context affects the *C*. *elegans* escape response, we applied localized thermal stimuli to similarly treated worms on bare plates and on plates seeded with an *E*. *coli* OP50 bacterial lawn, the standard laboratory food source for *C*. *elegans*. We confirmed previous reports [38,39], showing that baseline (pre-stimulus) velocity is higher off food (Fig 4C, *t* = -1 to 0 s), and that unstimulated worms off food spend less time reversing (Fig 4A and 4B, *t* = -2 to 0 s). We also observed that other characteristics of the baseline behavioral state distribution are significantly different between on food and off food conditions. When worms are off food, the probability of being in a forward state and the probability of turning are increased, while the probability of being in a pause state is lower (Fig 4A and 4B, *t* = -2 to 0 s).

When worms on food encounter noxious thermal stimuli, they respond more frequently and more robustly than worms off food (Fig 4A–4C). On food, more animals execute reversals in response to noxious heat, the reversals last longer (Fig 4B), and the average speed of escaping worms is higher than those off food (Fig 4C). The combined effect of these behavioral differences is reflected in the escape distance (Fig 4D), particularly in the four seconds immediately following the stimulus, in which animals on food escape further away from the stimulus. Eventually, the effect of the higher baseline speed of worms off food outweighs the faster and longer escape reversals of worms on food, and by eight seconds post-stimulus, worms off food are further away from the location of the noxious stimulus (Fig 4D). These results indicate a strong context dependence of the escape response, suggesting that worms integrate rapidly changing thermosensory measurements with information about their current food substrate when deciding how to respond to noxious heat.

### Escape strategy depends on initial behavioral state

**Fig 5:**
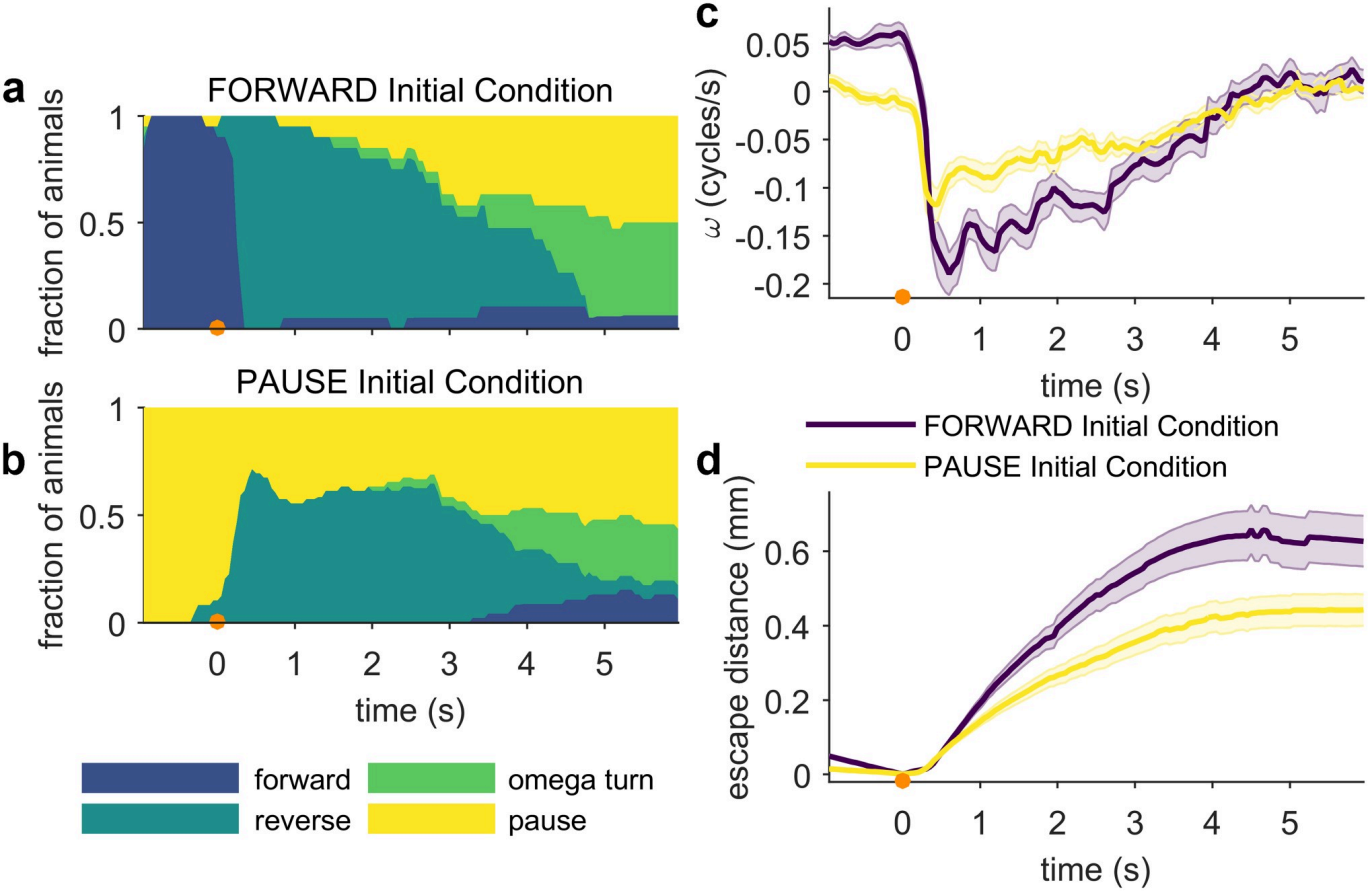
Sensorimotor processing and escape behaviors depend on the animal’s behavioral state prior to the stimulus. Stimulus (133 ms 95 mA, focused by 100 mm lens) is at *t* = 0 s (indicated by an orange circle). (a-b) Fraction of animals in each behavioral state, separated by initial condition (FORWARD n ≥ 15; PAUSE n ≥ 56). Note the much larger proportion of FORWARD initial condition animals that reverse in response to the stimulus, and the extended length of those reversals. (c) The population mean phase velocity, separated by initial behavioral condition. Animals moving forward before the stimulus are averaged in purple, animals that are paused in yellow. Shaded regions indicate the standard error of the mean at each time point. (d) Mean escape distance. Shaded regions indicate the standard error of the mean at each time point. The population of worms moving forward before the stimulus escapes significantly further. Full ethograms shown in S4 Fig.

It has been demonstrated that for some stimuli, the behavioral response depends on current body posture [6] or behavioral context [40]. To explore if this is true for thermal noxious escape, we analysed the pre-stimulus behavioral states of a large population of similarly treated worms, and compared the context-dependent escape responses of individuals that were paused at the time of stimulation with those that were moving forward. We found that a worm’s response to identical stimuli depends on its initial behavioral state when the stimulus was applied: animals that are moving forward when they encounter a stimulus almost always reverse (Fig 5A), whereas if an animal is paused when faced with noxious heat, it only reverses approximately 60% of the time (Fig 5B). Furthermore, animals moving forward prior to the stimulus exhibit much greater changes in velocity than the paused population (Fig 5C). These differences in velocity and reversal probability are reflected in the escape distances achieved by the forward-moving and paused animals: the population moving forward before the stimulus achieves significantly greater escape distances in as little as 1 s, and remains further away from the stimulus location for the duration of the assay (Fig 5D). Together, these results indicate a second type of state-dependence of the noxious heat response, in addition to the food-context-dependence described above; notably, behavioural responses to noxious heat depend on initial behavioral state.

### Escape reversals must complete before additional stimuli can evoke a response

Having established that whether a worm is paused or moving forward prior to encountering a noxious thermal stimulus changes the probability and magnitude of its escape response, we asked whether *C*. *elegans* is able to sense and respond to additional noxious stimuli while already executing an escape response, in order to broaden our understanding of this avoidance behavior’s state-dependence. To answer this question, we performed a “double zap” experiment, where we applied a second IR laser stimulus two seconds after the first. Most worms respond to the first stimulus by rapidly reversing. However, by two seconds after the stimulus, some animals have stopped reversing and entered a pause state (‘Short Response’). This distinction between ‘Short’ and ‘Long’ escape reversals has also been recently reported in response to sorbitol and optogenetic stimulation of ASH neurons [41].

**Fig 6:**
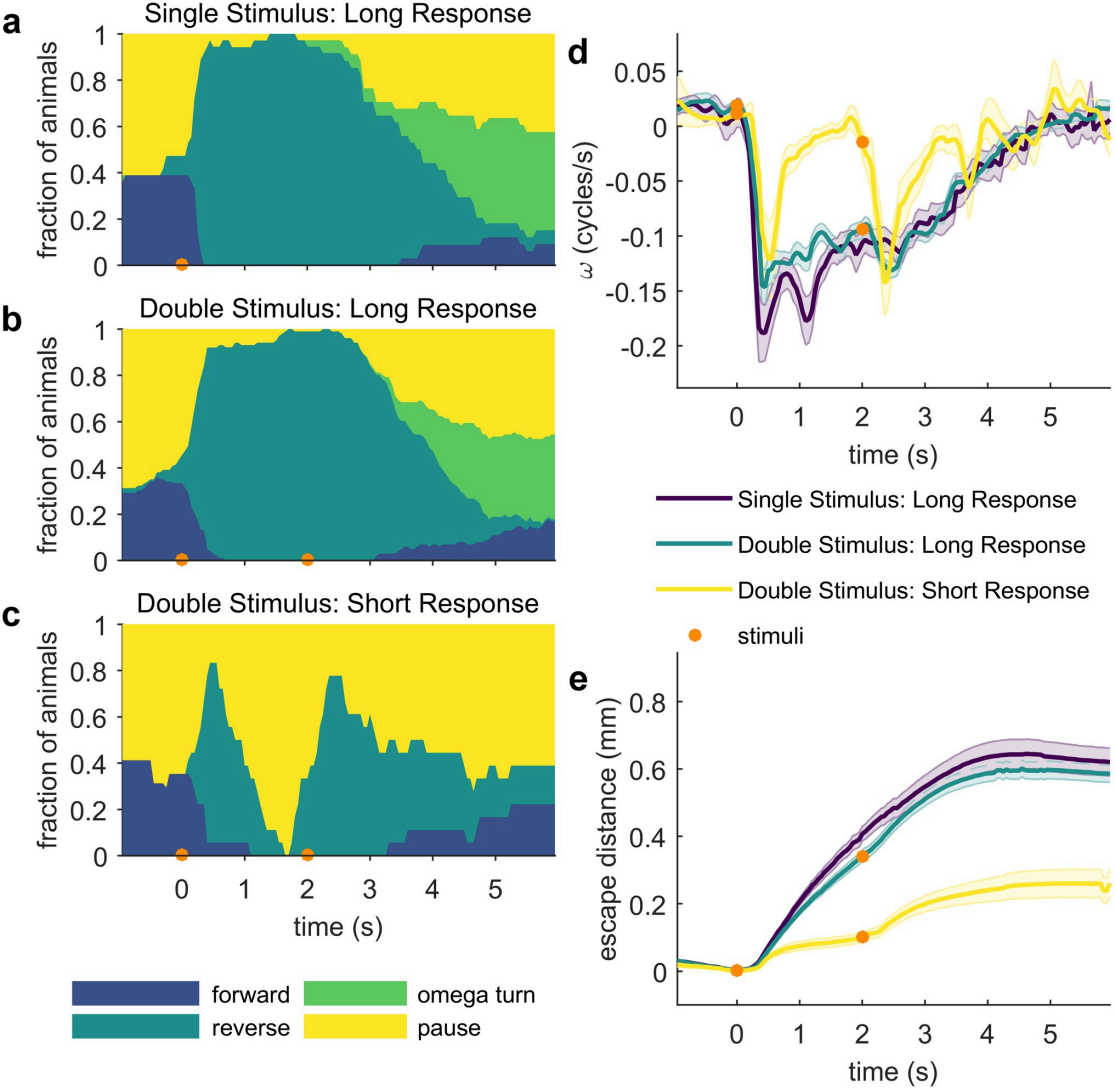
Initiation of the escape response is suppressed while an escape reversal is still underway. (a-c) Fraction of animals occupying each behavioral state in response to either a single (a) or double (b and c) thermal stimulus, separated by 2 s (133 ms 95 mA, focused by 100 mm lens). Only animals that respond to the first stimulus with a reversal are included. The SHORT and LONG responding animals are separated on the basis of the behavioral state occupied prior to the second stimulus, at 2 s after the first stimulus. Animals that have stopped reversing and transitioned into a paused state are categorized as SHORT response, while animals that are still reversing are labelled LONG. Note the similarity in the behavioral state distribution over time after the second stimulus between the single- (a) and double-stimulated (b) LONG responders. Stimuli timing is indicated with orange circles. Single stimulus n ≥ 32; double stimulus LONG n ≥ 74; double stimulus SHORT n ≥ 14. (d) Average phase velocity of worms in each of the three populations in (a-c). Shaded regions indicate the standard error of the mean. Note the robust response to the second stimulus from the SHORT responding population (d, yellow); also visible in (c). (e) Mean escape distance for each of the three populations in (a-c). Shaded regions indicate the standard error of the mean. Full ethograms shown in S5 Fig.

The population of worms that have completed their reversal response when the second stimulus is applied (‘Short Response’, Fig 6C) responds more robustly than worms still reversing (‘Long Response’), showing a change in speed that resembles their naive response (Fig 6D). In the ‘Long Response’ population, the proportion of animals in each behavioral state after the second stimulus closely resembles the behavioral distribution of the single stimulus population (Fig 6A and 6B). Similarly, there is no significant difference between the escape distances of ‘Long Response’ worms stimulated twice and worms stimulated only once (Fig 6E). Together, these results illustrate a second type of behavioral state-dependence for noxious heat avoidance: if a worm is executing an escape reversal, responses to additional noxious stimuli are repressed. In contrast, if the initial escape reversal is complete before encountering the second stimulus, a robust escape response can be executed.

### Response to thermal stimuli habituates after multiple encounters

**Fig 7:**
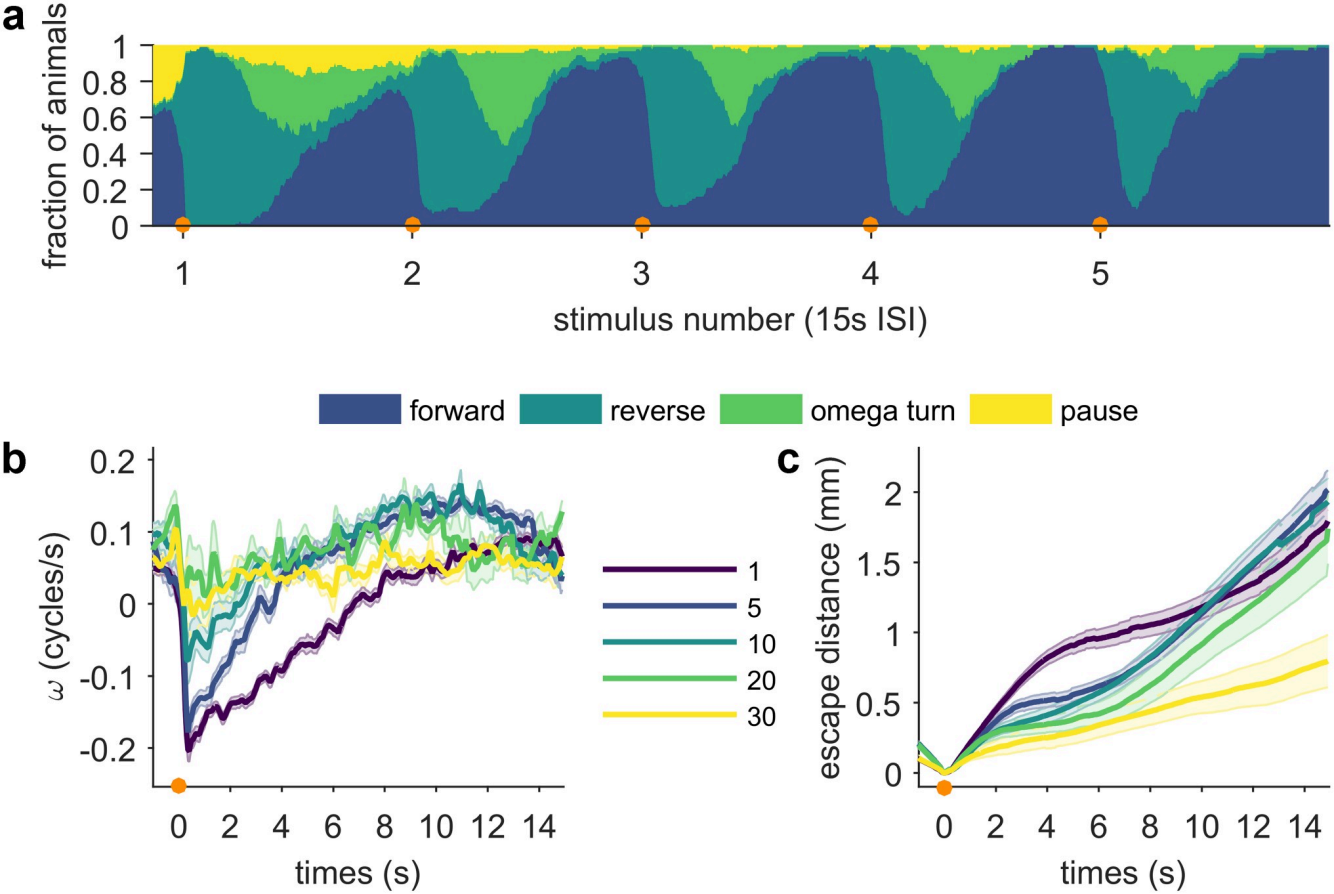
*C*. *elegans* sensory processing of thermal stimuli habituates with repeated encounters with a stimulus. IR laser stimuli (100 ms 75 mA, focused by 75 mm lens) were automatically targeted to the animal’s head every 15 s (indicated by orange circles). (a) Fraction of animals occupying each of the four behavioral states after repeated stimuli. Note the decreasing proportion of animals in the turn and pause state for the first several stimuli. n ≥ 14. (b) Mean phase velocity of animals that have been stimulated 1 (purple), 5 (blue), 10 (teal), 20 (green) and 30 (yellow) times. Shaded regions indicate the standard error of the mean. (c) Escape distance. Shaded regions indicate the standard error of the mean. Full ethograms shown in S6 Fig.

After showing that the escape response depends on an animal’s pre-exposure and stimulus-induced behavioral states, we asked how repeated encounters with noxious thermal stimuli affect *C*. *elegans* sensory processing over longer time periods. We tracked individual worms and applied stimuli to their heads every 15 s. This interstimulus interval (ISI) was chosen so that the sequence of immediate escape behaviors (reversal, turn, resumed forward motion) is completed by almost all animals before a subsequent stimulus is applied, and because it is within the range of ISIs to which *C*. *elegans* habituates its response to non-localized mechanical tap [42]. With repeated stimuli, the maximum escape reversal velocity decreases steadily (Fig 7B). Interestingly, this decrease in maximum reversal velocity does not closely resemble responses to lower power stimuli (Fig 3). That is, at lower power stimuli, while reversal speeds are lower, the more salient change is the decrease in reversal probability (Fig 3 and [16]). In contrast, with repeated stimuli at the same power, reversal probability remains high despite significant reductions in other aspects of the response, such as reversal velocity and duration (Fig 7A and 7B). These decreases of certain aspects of the escape response resemble the habituation to mechanical tap that has been reported [43]. However, the habituation we observed seems to be a more complicated phenomenon, with different features of the response decaying at different rates. Some characteristics, such as the velocity of the worm’s resumed forward motion after reorientation, increase after several subsequent stimuli, before later decaying (Fig 7B). Fig 7C illustrates how these changes in the escape responses to different numbers of stimuli affect how well *C*. *elegans* avoids the location of the stimulus. Surprisingly, large escape distances are achieved by 10 s post-stimulus in response to both early (stimulus 1, Fig 7E) and late stimuli (stimuli 5, 10, and 20, Fig 7E), but with divergent navigational strategies: large escape distances in response to early stimuli are achieved by extended, high velocity reversals, whereas large escape distances to later stimuli are achieved by sustained high velocity forward locomotion past the stimulus location, combined with shorter reversals (Fig 7A–7C).

### Long-term thermal experience modulates escape response

**Fig 8:**
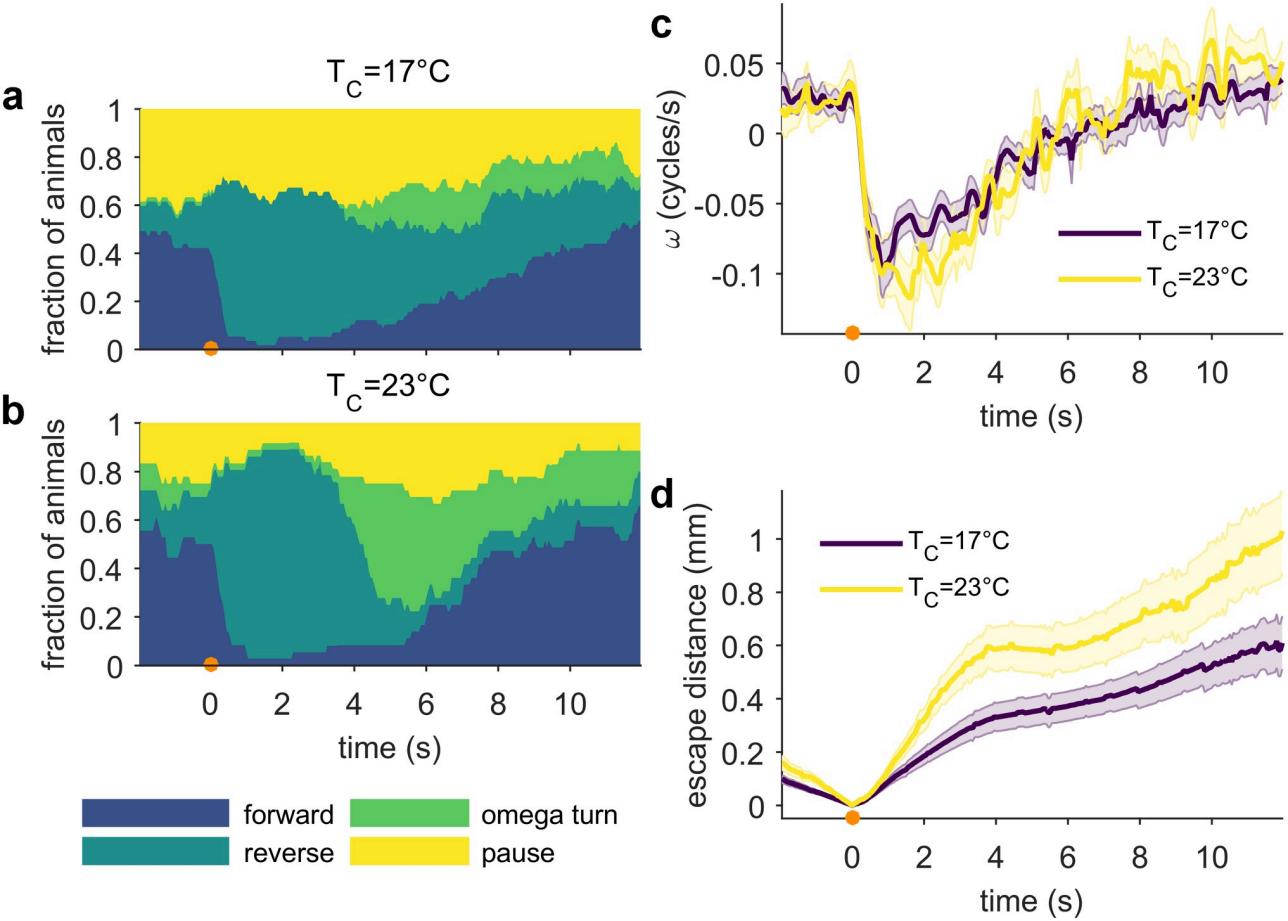
Animals cultivated at lower temperatures exhibit reduced escape responses. Animals were reared in incubators set to 17 °C and 23 °C. All behavioral experiments were performed at 23 °C. Stimulus (100 ms 75 mA, focused by 75 mm lens) is applied at *t* = 0 s. (a) and (b) show fraction of animals raised at each temperature in each of the four behavioral states after a thermal stimulus. n at 17 °C ≥ 53; n at 23 °C ≥ 32. (c) Mean phase velocity after stimulus. Shaded regions indicate the standard error of the mean. (d) Mean escape distance. Shaded regions indicate the standard error of the mean. Full ethograms shown in S7 Fig.

*C*. *elegans* thermosensory behavior is regulated, in part, by their previous temperature experience—animals migrate toward their cultivation temperature (*T*_C_) on thermal gradients, and track isotherms near *T*_C_ [22,44]. In the noxious range (29 °C to 37 °C), pre-exposure to high temperatures above *T*_C_ (1 hr at 28 °C) increases the threshold temperature required to elicit avoidance behaviors from worms on thermal gradients [45]. To determine whether past experience also modulates escape from rapidly increasing temperature, we examined the escape responses of worms reared at different temperatures. Surprisingly, when assayed at an ambient temperature of 23 °C, worms grown at 17 °C respond less frequently and less robustly than worms grown at 23 °C (Fig 8A and 8B). The average reversal velocity and omega turn probability is lower for *T*_C_ = 17 °C animals (Fig 8A–8C), and consequently the population of *T*_C_ = 23 °C animals achieves a greater distance away from the location of the noxious stimulus (Fig 8D). These results indicate that worms’ past temperature experience affects their behavioral response to acute noxious heat, suggesting that to execute an escape response, worms integrate rapidly changing thermosensory information with longer term memory of ambient temperatures.

## Discussion

We developed a new assay for interrogating the behavioral responses of *C*. *elegans* to noxious thermal stimuli. This assay enables: observing individual animals for long periods of time; high-resolution imaging of animal posture; and precise spatiotemporal control of a focused thermal stimulus with high anatomical specificity. We combined this experimental approach with comprehensive behavioral phenotyping, leveraging both continuous measurements, such as phase velocity and position, as well as discretized behavioral states, such as turns, pauses and reversals. The ability to apply precise time-varying thermal stimuli to individual animals and comprehensively quantify their behavioral responses revealed previously unknown complexity and extensive context-dependence in this apparently simple behavior.

By following individual animals for up to five minutes after they encountered a noxious thermal stimulus, we found that a single 100 ms thermal pulse is sufficient to induce elevated speeds and a shifted behavioral state distribution for many minutes, indicating a residual effect of the stimulus on that timescale. Investigators have shown *C*. *elegans* enters an arousal state characterized by increased speed when stimulated, such as when they are handled mechanically [46] or incubated at high temperature [47]. Our result parallels the recent finding that aversive mechanical stimuli lead to long-term locomotory arousal [48]. In that paradigm, locomotory and neuronal arousal last for one to two minutes and depends on FLP-20 neuropeptides from the touch receptor neurons (TRNs) and cell-specific activity of the FLP-20 receptor FRPR-3 in the RID neuron. Despite the similarity in the behavior, FLP-20 is not expressed in any known thermosensory neurons [49], which suggests that the increase in the worm’s locomotion might be regulated by independent mechanisms in different sensory modalities. Investigating the role of neuropeptide signalling in noxious heat-mediated locomotory arousal will be an interesting direction for future study.

The avoidance of noxious heat by *C*. *elegans* depends on integrating contextual cues about the animal’s local environment, such as food. We demonstrated that sensitivity to noxious thermal stimuli is reduced when well-fed animals are off food. With this finding, we add noxious heat to the other aversive stimuli the responses to which depend on food availability, including CO_2_, octanol and nose touch [50–52]. For octanol and nose touch, this sensitivity depends on dopamine and neuropeptide signalling in the polymodal nociceptor neurons ASH [53], however the neuronal signalling that underlies the food-dependent tuning of noxious heat sensitivity that we report here is unknown. That the presence of food significantly alters the *C*. *elegans* noxious heat avoidance strategy demonstrates that the worm is capable of rapidly integrating multiple sensory inputs when deciding how to respond to potentially harmful stimuli.

We investigated how an animal’s current behavioral state affects its sensorimotor response to noxious heat in two ways, by considering the influence of pre-stimulus behavioral state, and by applying a second stimulus to escaping worms. In both cases, we observed a strong state-dependence of *C*. *elegans* noxious heat avoidance behavior. An animal moving forward when it encounters a noxious stimulus is far more likely to respond than if it is paused, suggesting that the worm combines its current behavioral state with information collected by the thermosensory system. Why this behavioral strategy might have evolved is not immediately obvious. It is possible that the reason is purely sensory, since a faster moving worm will perceive a thermal stimulus as changing temperature at a higher rate than a stationary worm, and therefore determine that it is a more dangerous threat. Another possibility is that ecologically different strategies in threat response, depending on these small behavior differences in initial conditions, lead to different survivability outcomes.

When a worm encounters a second noxious thermal stimulus while it is still reversing away from the original stimulus, it appears to ignore the second stimulus, neither interrupting the first escape response to initiate a second, nor integrating the second stimulus and extending the first. This result suggests that the escape reversal has characteristics of an all-or-none response, which must finish before another escape reversal can be initiated. Recent projects exploiting optogenetic activation of specific mechanosensory neurons in *C*. *elegans* and descending neurons in the fruit fly *D*. *melanogaster* also revealed that an animal’s behavior immediately prior to a stimulus can bias its behavioral response [40,54]. The results of those optogenetic interrogations, combined with the evidence presented here that the noxious heat avoidance response is strongly behavioral state-dependent, suggest that this type of context-dependency may be generalizable across various sensory modalities and evolutionarily conserved between phyla. How the *C*. *elegans* nervous system encodes information about its current behavior, and how it integrates this information with incoming sensory stimuli remains unclear. We anticipate that our system for precisely applying dynamic thermal stimuli and comprehensively quantifying behavior will prove useful for answering these questions.

In addition to current behavioral state, we found two ways that the worm’s temperature experience modulates noxious heat avoidance: first, the escape response depends on the animal’s long term thermal history (cultivation temperature), and second, worms reduce but never eliminate avoidance behaviors after repeated encounters with noxious thermal stimuli. Previous work has revealed that sensed temperature experience can impact longevity [55], high temperature thermal tolerance [56], and noxious heat avoidance responses [45]. Our results add to the wide range of behaviors that reflect the worm’s thermal memory, and emphasize how important storing a record of thermal experience is for the worm.

We have demonstrated that *C*. *elegans* implements a diverse range of behavioral strategies to escape from noxious heat. Worms are able to tune their escape responses to various threat levels, combine inputs from distinct sensory pathways to incorporate food context in their decision making, and modulate their escape responses in an experience and behavioral state dependent way. Remarkably, the small nervous system of the worm simultaneously integrates memory of long-term thermal experience on the timescale of days with information about its immediate behavioral state on the timescale of seconds. With this work we have placed the noxious heat escape response in a broader context than previously considered, and in doing so have revealed surprising complexity and plasticity in a well studied behavior. The noxious thermal response assay described here will be useful for future investigations of how such a simple circuit in *C*. *elegans* can rapidly integrate current behavior with multiple sensory inputs to execute a complex escape response with several degrees of freedom.

## Materials and methods

### Strain cultivation

*C*. *elegans* Bristol N2 hermaphrodites were cultivated at 23 °C (except when otherwise indicated) on standard nematode growth medium (NGM) plates seeded with *E*. *coli* OP50 following standard cultivation methods [57].

### Applying repeated thermal stimuli to freely moving animals

#### Automatic tracking and thermal stimulation

We integrated a steerable infrared (IR) laser with a worm-tracking microscope. A 1440 nm diode laser (FOL1404QQM; Fitel, Peachtree City, GA) is steered by a pair of galvanometers (GVS002 TSH24006 X/Y; Thorlabs, Newton, NJ), reduced in intensity as necessary by ND filters and focused to spot by a 75 mm or 100 mm focal length lens. The diode is driven and temperature controlled by a Thorlabs diode controller (LDC 240C) and temperature controller (TED 350). A polarized beam-splitter is used to reflect the beam to the surface of the assay plate and enables imaging from above. Assay plates are illuminated from below with red light from an adjustable output LED (DiCon; Richmond, CA). Images were captured at 15–20 Hz by a digital camera (MANTA 125B ASG; Allied Visions, Stadtroda, Germany), magnified by a variable magnification imaging lens (55–906 MMS R-2; Edmund Optics, Barrington, NJ). Custom machine vision software written in LabVIEW (National Instruments, Austin, TX) evaluates the worm’s location in each frame and updates the position of the XY tracking stage controlled by a 2-channel stepper motor controller (BSC-102; Thorlabs) in order to keep the worm in the field of view. Simultaneously, the program processes each frame to isolate a binary image of the worm and identify the location of the head and tail to allow precise stimulation with the laser. Combined tracking and thermal stimulation is possible up to 20 Hz. The duration of tracking and number of stimuli is limited only by disk space. The accuracy of the laser stimulus position relative to the target position on the worm is limited by the worm’s displacement between the time the stimulus is applied and the time at which the frame immediately prior was recorded, which is used to calculate the target position. Our system allows full control of the duration, frequency, intensity and position of the thermal stimulus. For the experiments described in this work, two stimulus regimes were used: (1) the animals represented in Figs 2, 3, 4, 7 and 8 were stimulated with a 100 ms pulse focused by a 75 mm lens (37.5 mA, 75 mA or 375 mA, as indicated). (2) the animals represented in Figs 5 and 6 were stimulated with a 133 ms 95 mA pulse focused by a 100 mm lens, in order to facilitate comparison with other studies [16,58,59].

#### Behavioral assays

All behavioral assays except for the food context experiment (Fig 4) were performed on assay plates with food. Assay plates were prepared for the thermal stimulus assay using a previously developed protocol [15, 16]. 16–24 hours before testing, assay plates were seeded with 100 μL *E*. *coli* OP50 (OD_600_ = 0.6–0.8), and stored at room temperature overnight. Unless otherwise indicated, young adult worms were assayed. Between 1–10 animals were picked to each assay plate. For single-animal experiments, the dorsal-ventral orientation was annotated by eye when each worm was picked to its assay plate. We assumed that worms did not flip themselves through the course of the experiment. The underside of the plate lid was polished with a drop of detergent to prevent collection of condensation interfering with imaging. All behavioral experiments were performed in a temperature controlled room set to 23.0 °C. Three timing protocols were used: (1) for experiments on food, worms were allowed to recover from picking for at least 30 minutes at 20 °C or 23 °C. (2) For experiments off food and relevant controls, worms were washed twice in M9 buffer, picked to an unseeded plate, and allowed to recover from picking for 10 minutes before assaying, and then experiments were completed within the next 30 minutes. This time frame was used to limit possible changes in behavior due to extended periods of time off food. (3) For the 2 second ISI double-stimulus and initial condition dataset (Figs 5 and 6), worms were washed twice in M9, gently spun down for 1 minute, then incubated in M9 for 30 minutes. The worms were then transferred to an assay plate, and allowed to recover from picking for 30 minutes. Trials were then completed within the next 30 minutes. This protocol was used to make quantitative comparison with other datasets possible [16,58,59].

### Data analysis

All offline image processing and behavioral analysis was completed in MATLAB (R2015a, The Mathworks, Natick, MA).

#### Image segmentation

First, worm bodies are segmented from the background. Because imaging conditions are variable across different experiments, a set of histogram-based binary thresholding algorithms are polled to find a threshold value that best segments each image. This segmented image is used as a seed mask for the Fast Marching Method, to generate a final segmentation [60]. Holes in the worm body are filled, except for holes which fulfill selection criteria matching the centre of a looped worm.

#### Calculation of worm skeletons

Worm skeletons are generated in two primary steps. First, a skeleton for each frame is calculated with a robust midline-finding algorithm described previously [15, 58]. This algorithm works very well for frames with simple worm postures, but fails during self-occluding coils and deep turns. Frames with crossed worms or binary segmentation failures are flagged using skeleton length and worm shape characteristics, and are double-checked with the user. In the second step, skeletons for these frames with complex worm shapes are calculated using an eigenworm-based posture-tracking algorithm [11]. The final step of this algorithm interpolates across time through posture space, which ensures that the best solution for each crossed frame is found, and also reduces minor errors in simple, non-overlapping frames.

#### Head-tail correction

The skeleton tracking algorithm described above occasionally reverses the head and tail of the worm. To accurately and efficiently assign the head and tail, we exploited the location of each stimulus, which is known to be at the head. Moving forward and backward from each stimulus frame, the skeletons were checked and flipped as necessary, to minimize the change in head-position from frame to frame. In the case of segmentation errors which interrupt this process, the user is asked to annotate the head in a single frame after the error, and the correction process continues.

#### Coordinate systems

Before each recording, the XY stage is calibrated by tracking the movement of a stationary object on the plate (a speck of dust, for example), in order to determine the transformation matrix to convert pixel coordinates to real-world coordinates. The laser galvanometers are also calibrated, by asking the user to click on the position of the beam as it burns a spot on the surface of the agar plate. The beam is then steered by both mirrors and the new position is recorded, confirming the conversion between input voltage and pixel-movement.

#### Stage movement estimation

To measure the real-world position of the stage in each frame, including frames during which the stage is in motion, we calculated the frame-frame movement of the tracking stage using an image-registration based strategy. The translation between each pair of frames is calculated using a fast subpixel image-registration algorithm which utilizes the discrete Fourier transform [61]. A region from the edge of each frame where no worm is present is used, to avoid attempted registrations of the worm’s body, which might change position frame to frame. The pairwise translations were averaged with translations between frames 2 and 3 frames apart, to reduce the effect of accumulated noise in summing across many frames. The cumulative translation between frames is converted from pixels to mm (stage units), and added to the starting position of the stage, as reported by the controller. These stage positions are used to calculate the real-world position of the worm and laser position in each frame.

#### Calculating translational velocity

The centroid of the binary image of the worm in each frame, converted to real-world units (mm), is used to calculate the magnitude of the worm’s translational velocity (speed). The x and y position vectors are first smoothed with a robust discretized spline smoothing algorithm [62,63] to reduce the effect of high frequency noise on the derivative calculation. The worm’s speed is the magnitude of this derivative. The worm’s heading (sign of the translational velocity), is assigned by taking the angle between the direction of the worm’s centroid movement for each frame [ctrxi—ctrx_i-1_, ctryi—ctry_i-1_], and the worm’s orientation, which is defined as the vector from the centroid to the worm’s head, [headxi—ctrx_i_, headyi—ctry_i_]. The translational velocity is positive (forward movement) for θ < π/2, and negative (reverse movement) for π/2 < θ < π.

#### Calculating phase velocity

The worm’s phase (*φ)* was described using the projections of the worm’s skeleton (modes *a_1_* and *a_2_*) on to the first two eigenworms, which dominate the worm’s posture during forward and backward sinusoidal motion, as described previously [6]. The modes are smoothed across time and posture space during the final interpolation step of skeleton calculation so are not smoothed further [11]. Phase is defined as *φ* = tan^-1^(−*a_2_*/*a_1_*). The phase velocity (*ω*) is then calculated by taking the derivative of this phase vector, *dφ/dt*. In some postures, when modes *a_1_* and *a_2_* are near zero, high frequency noise dominates the phase derivative. Therefore, we removed frames that resulted in physiologically impossible changes in phase velocity (*dω*/*dt*>0.18 cycles/s). Peaks in *dω*/*dt* were identified with the MATLAB *findpeaks* function, using a threshold of three standard deviations from the mean of dω/dt, calculated from a population of worms. Frames within half the peak width before and after each peak were removed from the phase velocity vectors. Peaks were generally no more than a few frames, less than 0.25 s. Positive phase velocities correspond with forward motion, and negative phase velocities correspond with backward motion. It is important to note that accurate calculation of direction depends on accurate labelling of the worm’s head in each frame.

#### Behavioral flagging

Four behavioral states are automatically flagged based on the worm’s skeleton and phase velocity: forward, reverse, pause and deep turn. Worms cannot occupy more than a single behavioral state simultaneously. The minimum duration of a behavioral segment is 0.25 s (5 frames). Fluctuations shorter than 0.25 s are assigned the state of the surrounding behavior. Forward and reverse behavioral flags are assigned according to the sign of the worm’s instantaneous phase velocity. Deep turns are flagged when the head-tail distance is less than half the average head-tail distance. Pauses are flagged when the magnitude of the phase velocity is less than 0.02 cycles/s. Behavioral flags are human-verified.

### Temperature measurement of infrared laser pulses

The local changes in temperature induced by the IR laser stimulus were measured using the pH sensitive dye BCECF [64] in combination with TRIS buffer, the pH of which is sensitive to temperature [65]. We assume that the agar and the boundary water layer dominate the thermal dynamics of the heating.

The infrared laser stimulus used in the behavioral experiments was replicated on top of an inverted microscope set up for dual-excitation imaging (Eclipse Ti; Nikon). The power of the laser was matched to the behavioral set-up using a power meter and IR-sensitive sensor (PM100D and S122C; Thorlabs), and an identical collimator and lens was used to focus the laser to the surface of the sample.

As in the behavioral assay, the IR laser was focused to the sample’s surface from above, and the top surface was exposed to air. We used an agar sample (10 mM TRIS buffer, pH 7.1) roughly 1 mm thick, made by stacking a 200 μm thick, surface layer with 5 μL 200 μM BCECF acid (85138-49-4; Biotium) in 10 mM TRIS (pH 7.1) and a bottom layer with no dye. The sample was mounted on a coverslip, surrounded by a custom aluminum stage with an integrated thermoelectric cooler (MCTE1-19913L-S; Farnell), the temperature of which was stabilized with a recirculating chiller (T255P-3CR; Coherent) and controlled by a benchtop controller (5R6-900; Oven Industries). The sample was sealed above with a piece of plastic cut from a petri dish lid, so that the laser passes through the same materials as in the behavioral experiments.

The surface of the sample was imaged from below using a high numerical objective with sufficient working distance to image through the ~1 mm sample (S Fluor 10x NA 0.5 WD 1.2; Nikon). The sample was excited by a high-power LED and driver (M470L2 and LEDD1B; Thorlabs), through either a 440 nm or 490 nm excitation filter. Emission at 535 nm was captured by an EMCCD (DU-897E-CSO-BV; Andor). Image capture was controlled by custom MATLAB software [66].

For each sample, the relationship between the change in the BCECF intensity ratio (490 nm / 440 nm excitation) and the change in temperature was calibrated by monitoring fluorescence from each channel at known temperatures (23 °C—26 °C). To help control for possible changes in dye distribution and concentration due to diffusion between the agarose disks or evaporation, each sample was calibrated before and after laser stimulation. Sample temperature at various set temperatures was measured separately using a thermocouple positioned at the surface of the agarose disk (5TC-TT-T-40-36; Omega).

Laser pulses at different power levels were applied to the sample every 6 s. For each sample and power level, a number of pulses [20–50] were aligned in time and averaged. We acquired one frame from the temperature insensitive channel at the beginning and end of each laser pulse experiment, and captured the temperature sensitive channel at maximum high frame rate (~20 fps). The temperature insensitive signal was interpolated between the start and end points as previously described [67].

For each set of aligned pulses, the difference in the ratio of BCECF intensities (490 nm / 440 nm excitation) between each frame of the pulse time series and an average of 3 pre-stimulus frames was calculated. The Δratio/Δtemperature relationships obtained from calibrating each sample were averaged and used to calculate the change in temperature above baseline for the full field of view at each time-point. To estimate the maximum temperature, a 60 pixel diameter (37.4 μm) circular region of interest (ROI) was averaged around the center of the beam. Image sequences were processed with custom programs written in MATLAB.

## Supporting information

S1 Fig Spatial and temperature characteristics of infrared laser stimulus. (a) Distribution of head-stimulus distances. The euclidean distance between the first skeleton point at the head end of the worm and the galvanometer positions in the first frame of the stimulus. (b) The temperature of the surface of an agarose disk was measured using the pH sensitive dye BCECF in the temperature-sensitive buffer TRIS. The disk was made with 10 mM TRIS (pH 7.1) and treated with 5 uL 200 mM BCECF dye in 10 mM TRIS (pH 7.1). The spatial temperature profile of the 375 mA pulse as it decays from its peak is shown. Numerous individual pulses (>20) from 6 samples were combined, and then radial profiles from the center of the pulse were averaged. Shaded regions indicate the standard error of the mean. (c) The local temperature at the focus of the beam increases rapidly with laser onset, and decays back to baseline over the course of ~2.5s. Four laser current levels are shown, including the three used in this paper. Shaded regions indicate the standard error of the mean. (d) Peak pulse temperature increases linearly with laser current. Error bars show the standard error of the mean; for 37.5 mA and 75 mA the values are smaller than the data markers.
(TIF)

S2 Fig Stimulus amplitude behavioral ethograms. (TIF)

S3 Fig Food condition behavioral ethograms. (TIF)

S4 Fig Initial condition behavioral ethograms. (TIF)

S5 Fig Double stimulus behavioral ethograms. (TIF)

S6 Fig Habituation behavioral ethograms. (TIF)

S7 Fig Cultivation temperature behavioral ethograms. (TIF)
