# Supplementary figures and images for: Targeted thermal stimulation and high-content phenotyping reveal that the C. elegans escape response integrates current behavioral state and past experience
Source: PLoS One. 2020 Mar 27;15(3):e0229399. doi: 10.1371/journal.pone.0229399 (PMC7100941; doi:10.1371/journal.pone.0229399)

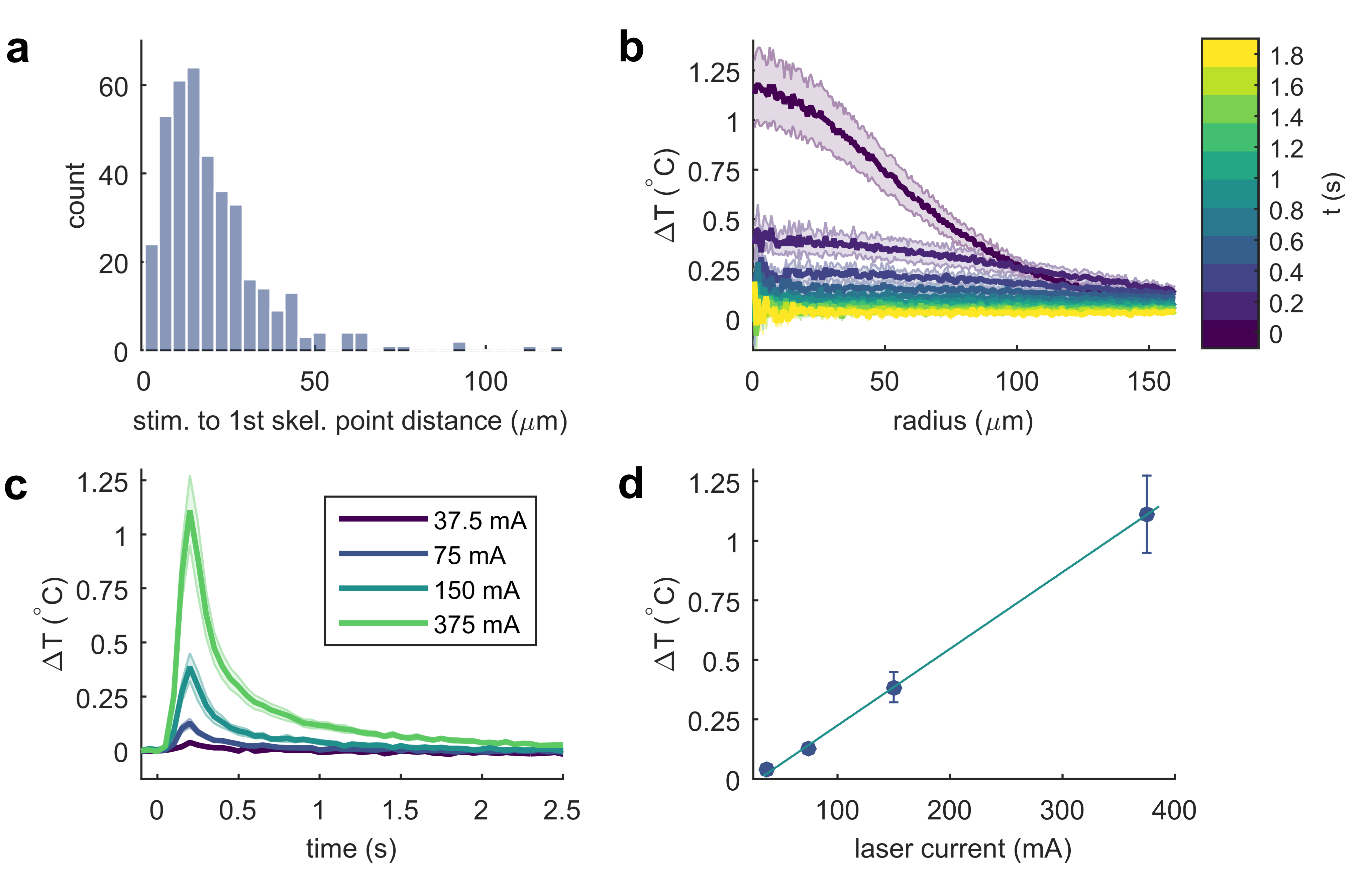

Supplement: S1 Fig — (a) Distribution of head-stimulus distances. The euclidean distance between the first skeleton point at the head end of the worm and the galvanometer positions in the first frame of the stimulus. (b) The temperature of the surface of an agarose disk was measured using the pH sensitive dye BCECF in the temperature-sensitive buffer TRIS. The disk was made with 10 mM TRIS (pH 7.1) and treated with 5 uL 200 mM BCECF dye in 10 mM TRIS (pH 7.1). The spatial temperature profile of the 375 mA pulse as it decays from its peak is shown. Numerous individual pulses (>20) from 6 samples were combined, and then radial profiles from the center of the pulse were averaged. Shaded regions indicate the standard error of the mean. (c) The local temperature at the focus of the beam increases rapidly with laser onset, and decays back to baseline over the course of ~2.5s. Four laser current levels are shown, including the three used in this paper. Shaded regions indicate the standard error of the mean. (d) Peak pulse temperature increases linearly with laser current. Error bars show the standard error of the mean; for 37.5 mA and 75 mA the values are smaller than the data markers. (TIF) [file pone.0229399.s001.tif]

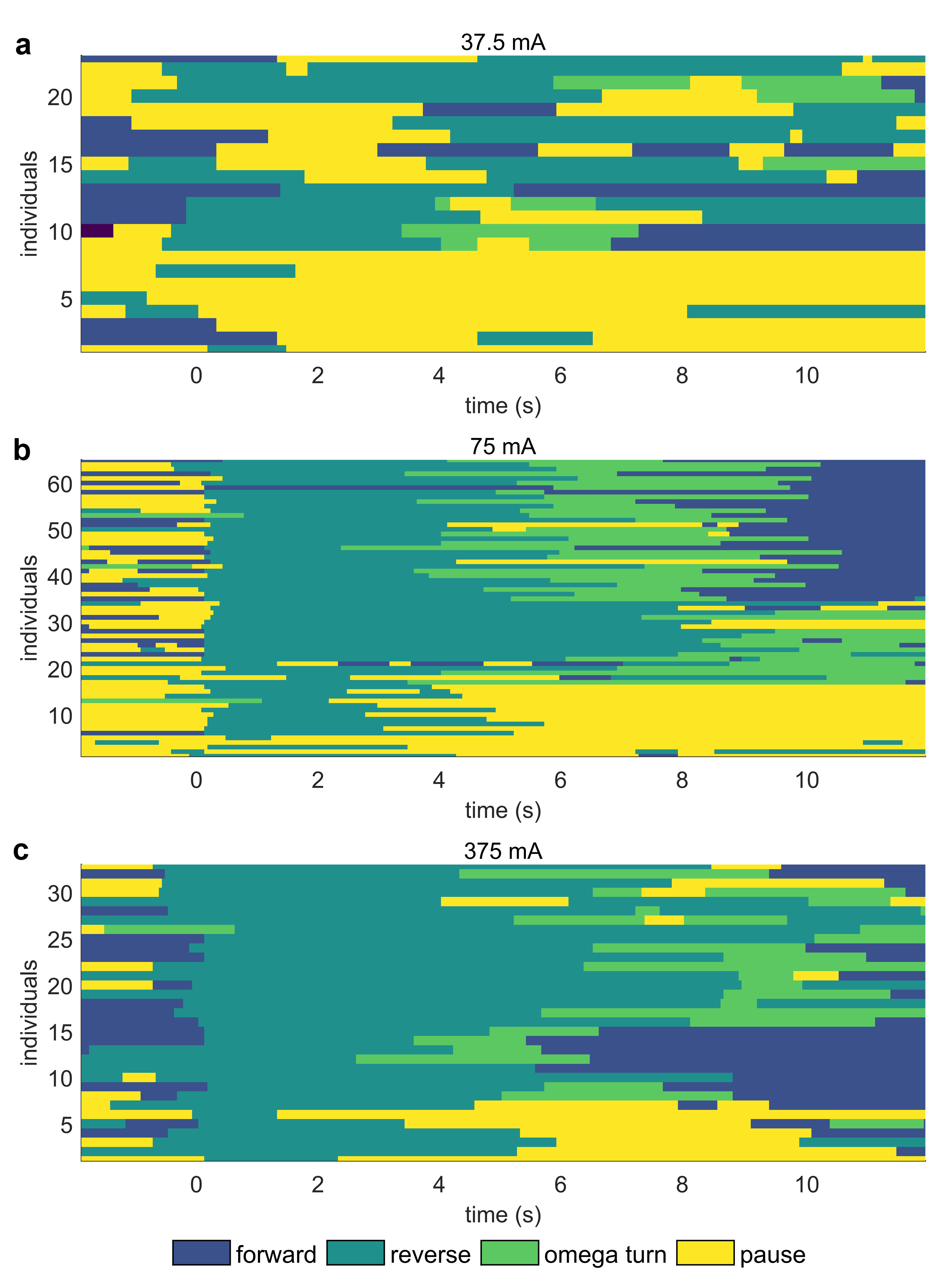

Supplement: S2 Fig — (TIF) [file pone.0229399.s002.tif]

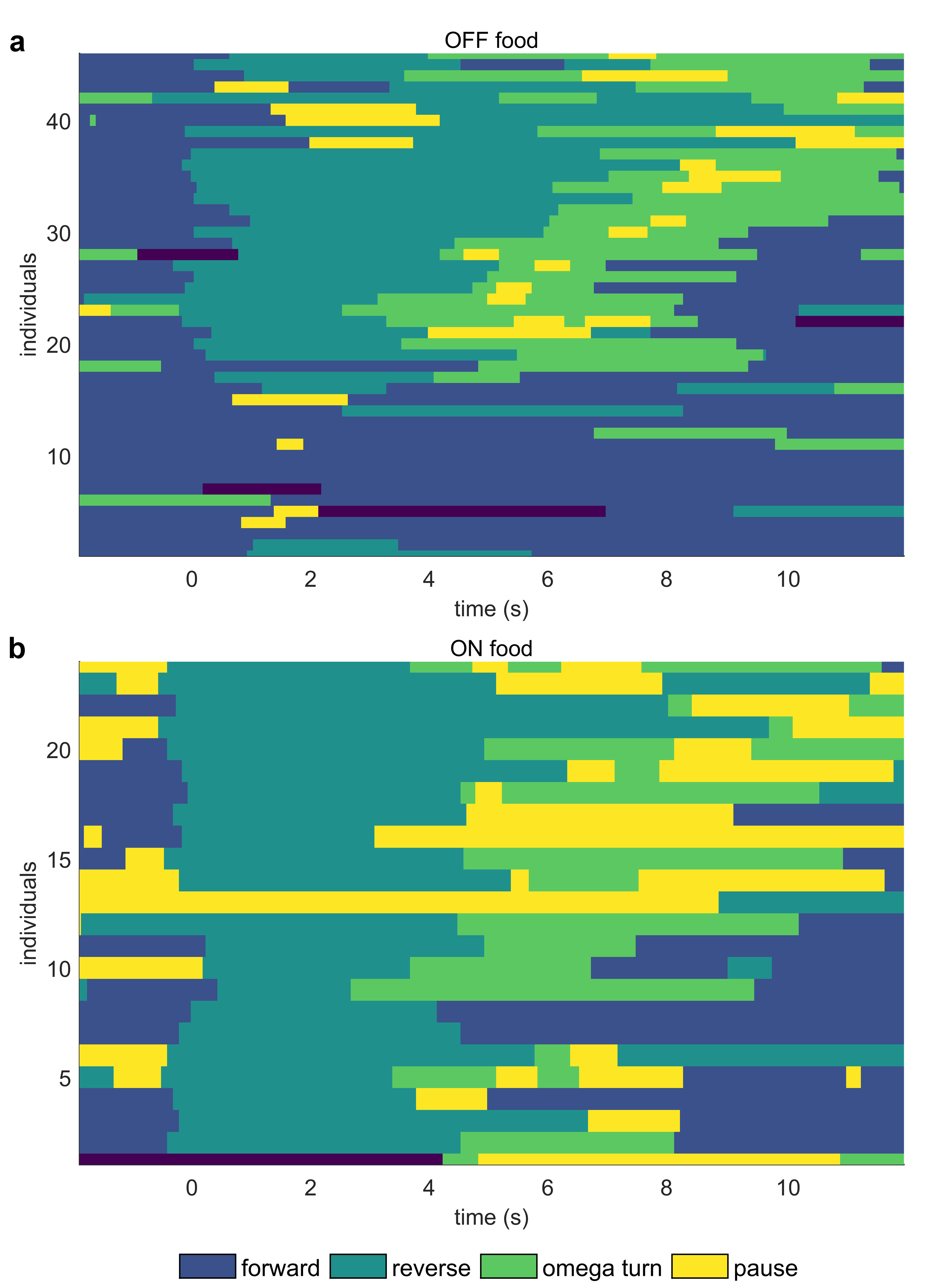

Supplement: S3 Fig — (TIF) [file pone.0229399.s003.tif]

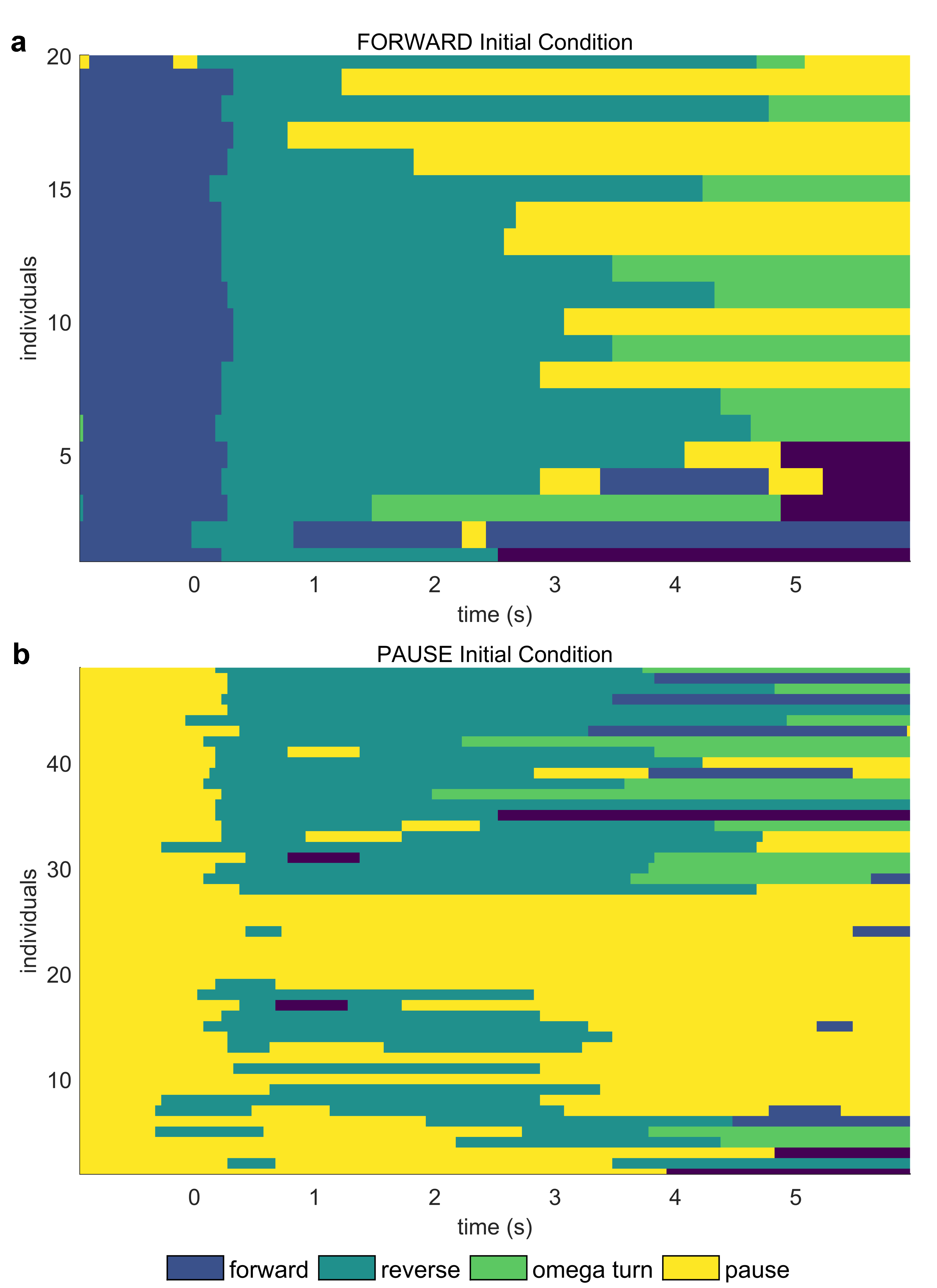

Supplement: S4 Fig — (TIF) [file pone.0229399.s004.tif]

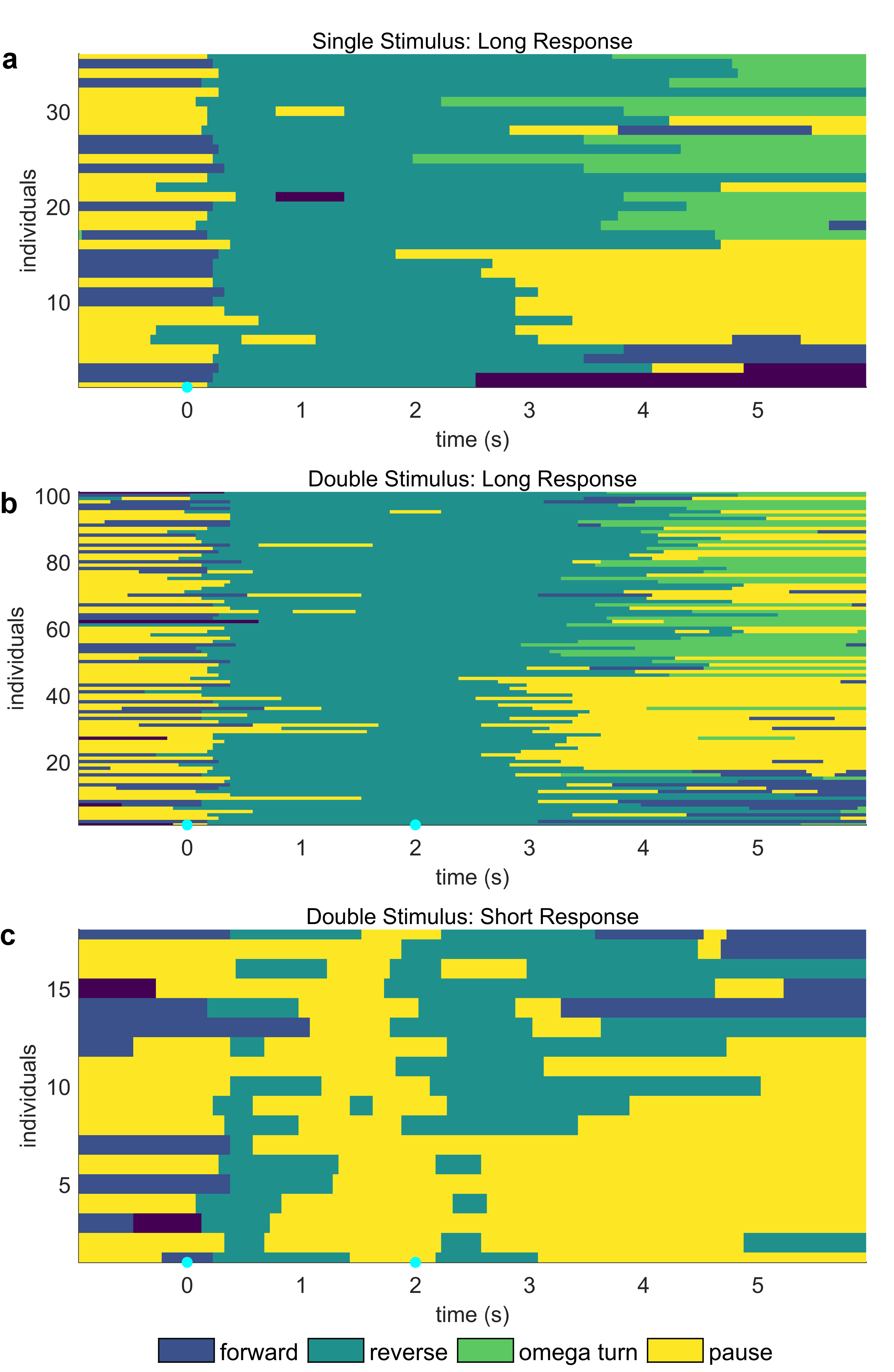

Supplement: S5 Fig — (TIF) [file pone.0229399.s005.tif]

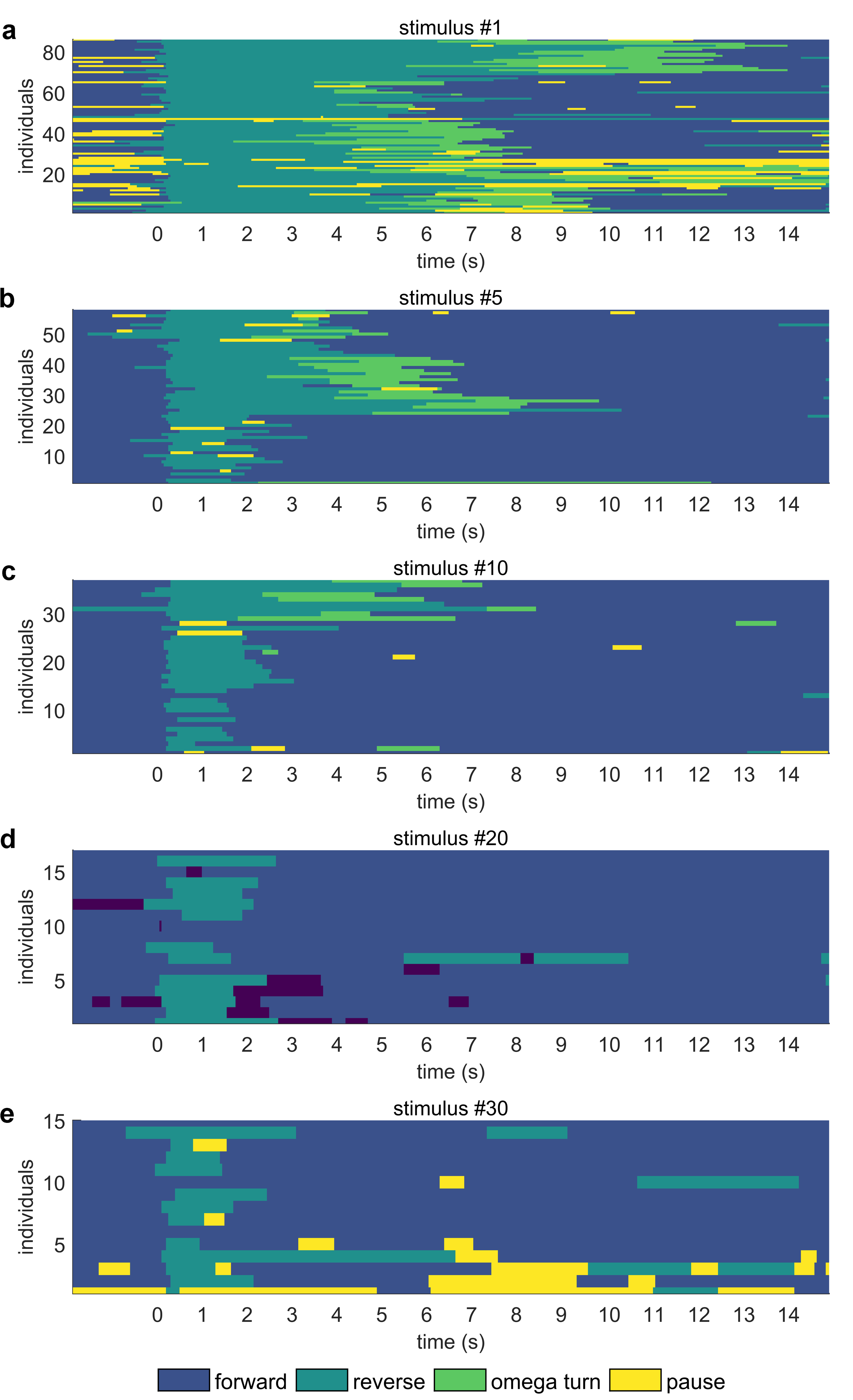

Supplement: S6 Fig — (TIF) [file pone.0229399.s006.tif]

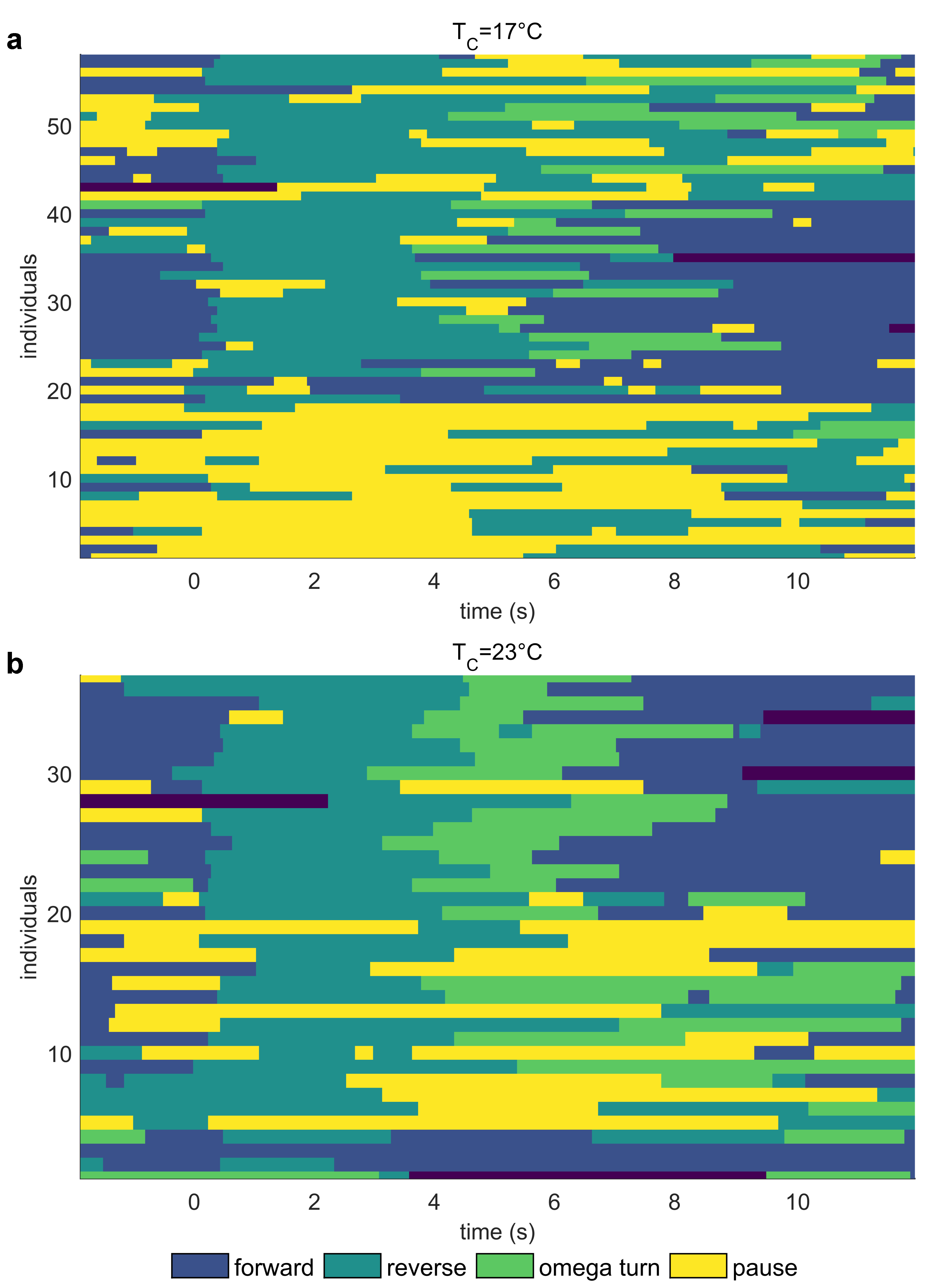

Supplement: S7 Fig — (TIF) [file pone.0229399.s007.tif]
